# Effects of exercise on clinical outcomes in patients undergoing haemodialysis for chronic kidney disease: an umbrella review

**DOI:** 10.7189/jogh.16.04227

**Published:** 2026-07-24

**Authors:** Meihui Huang, Zhihao Xie, Bin Lin, Wenfeng Xiao, Ting Wen, Yusheng Li, Haitao Long, Xucheng Yang, Kai Zhang

**Affiliations:** 1 Department of Orthopaedics, Xiangya Hospital, Central South University, Changsha, Hunan, China; 2 National Clinical Research Centre for Geriatric Disorders, Xiangya Hospital, Central South University, Changsha, Hunan, China; 3 Xiangya School of Medicine, Central South University, Changsha, Hunan, China; 4 Department of Orthopaedic Surgery, China-Japan Friendship Hospital, Chinese Academy of Medical Sciences and Peking Union Medical College, Peking, China; 5 Changde Hospital, Xiangya School of Medicine, Central South University, The First People’s Hospital of Changde City, Changde, China

**Keywords:** intradialytic exercise, haemodialysis, chronic kidney disease, umbrella review, aerobic exercise, resistance training

## Abstract

**Background:**

Exercise is increasingly recommended for haemodialysis patients with chronic kidney disease, yet the evidence from meta-analyses remains fragmented, and its methodological quality has not been systematically evaluated.

**Methods:**

We systematically searched Embase, PubMed/MEDLINE, Cochrane Library, and Web of Science up to January 2026. We evaluated the methodological quality of studies using the ‘Measurement Tool to Assess Systematic Reviews 2’. We used GRADE system to rate the evidence level for each outcome as high, moderate, low, or very low.

**Results:**

We reviewed 11 meta-analyses. Aerobic exercise significantly improved dialysis adequacy (Kt/V) (weighted mean difference (WMD) = 0.08; 95% confidence interval (CI) = 0.00, 0.15), cardiorespiratory fitness (VO_2_ peak) (WMD = 2.07; 95% CI = 0.42–3.72), six-minute walk test (6MWT) (WMD = 64.98; 95% CI = 43.96–86.11); systolic blood pressure (SBP) (WMD = –10.07; 95% CI = –16.35, –3.78), C-reactive protein (CRP) (WMD = –3.28; 95% CI = –4.68, –1.88), and depression (standardised mean difference = –0.93; 95% CI = –1.32, –0.55). Aerobic exercise did not significantly affect diastolic blood pressure (DBP), physical component score (PCS), mental component score (MCS), or serum phosphorus. Resistance exercise significantly improved 6MWT (WMD = 68.50; 95% CI = 29.05, 107.96) and PCS (mean difference = 10.05; 95% CI = 2.95, 17.14) with no significant effects on Kt/V, CRP, or depression. Combined exercise improved VO_2_ peak, DBP, PCS, MCS, CRP, and depression, but not Kt/V, 6MWT, or SBP.

**Conclusions:**

Aerobic exercise may improve cardiorespiratory fitness, dialysis efficiency, inflammation, and depression; resistance may enhance muscle strength and physical function; combined training offers the broadest benefits. Individualized prescriptions are supported, but high-certainty evidence is lacking.

**Registration:**

PROSPERO: CRD420251244373

Chronic kidney disease (CKD) is one of the leading causes of death and suffering in the 21st century, affecting over 10% of the global population – more than 800 million people [1–3]. End-stage renal disease (ESRD) is the final stage of CKD [4]. Haemodialysis is the predominant renal replacement therapy for ESRD patients [5]. In 2010, approximately 2.62 million patients received renal replacement therapy worldwide, and the number is expected to double by 2030 [6].

Physical inactivity is a major problem for patients with ESRD. Haemodialysis is a time-consuming and expensive treatment that imposes additional dietary and fluid restrictions. Inflammation-induced abnormal protein metabolism can exacerbate malnutrition, leading to decreased muscle mass, reduced exercise tolerance, and impaired quality of life [7–12]. This may be exacerbated by cardiovascular and pulmonary comorbidities, anaemia, hypervolemia, and fatigue following dialysis treatment [9,13]. In 2019, the clinical practice guideline for renal rehabilitation recommended exercise therapy for patients undergoing haemodialysis with a grade of 1B, indicating a strong recommendation supported by moderate-quality evidence [14]. In response, a growing number of meta-analyses and RCTs have synthesised the effects of exercise interventions in this population.

Existing meta-analyses have reported benefits of exercise across several clinical and patient-reported outcomes, but findings vary by exercise modality and outcome domain. For dialysis adequacy (Kt/V), some meta-analyses have reported significant improvements with intradialytic aerobic exercise, while others have found no effect [15,16]. For cardiorespiratory fitness (VO_2_ peak) and functional capacity, aerobic, resistance, and combined training have each shown positive effects, though the magnitude of benefit differs across reviews [17]. Health-related quality of life has improved inconsistently, with some meta-analyses reporting benefits only in the physical domain, while others show no significant change [18,19]. Regarding inflammatory markers, particularly C-reactive protein (CRP), several meta-analyses have demonstrated significant reductions following aerobic or combined exercise, but null findings have also been reported [20].

Although numerous meta-analyses have been conducted, several critical gaps remain. First, findings across meta-analyses are often inconsistent, even for the same outcome and exercise modality, due to differences in inclusion criteria, search dates, and analytical methods. Second, the methodological quality of meta-analyses has never been systematically appraised using a validated tool such as Measurement Tool to Assess Systematic Reviews 2 (AMSTAR 2), leaving readers uncertain which reviews are trustworthy. Third, the degree of primary study overlap among meta-analyses has not been quantified, which can lead to overestimation of precision if multiple reviews include the same original trials. Fourth, no previous overview has graded the certainty of evidence for each exercise-outcome pair using the GRADE framework, which is essential for clinical recommendations. Fifth, it remains unclear whether aerobic, resistance, or combined exercise modalities offer distinct, overlapping, or synergistic benefits for specific outcomes, as prior meta-analyses have often focused on a single modality or pooled them without stratification.

Given the fragmentation and inconsistencies in the existing evidence, a comprehensive synthesis that critically appraises methodological quality, quantifies overlap, and grades certainty by exercise modality is urgently needed. Therefore, we aimed to critically appraise the methodological quality of existing meta-analyses using AMSTAR 2, quantify primary study overlap using the Graphical Representation of Overlap for OVErviews (GROOVE) tool, grade the certainty of evidence for each exercise-outcome pair using GRADE, and provide a clinically actionable, modality-specific synthesis to guide exercise prescription in patients undergoing haemodialysis for CKD.

## METHODS

We systematically evaluated and synthesised evidence from multiple meta-analyses investigating the effects of exercise interventions on outcomes in patients with CKD undergoing haemodialysis [21,22]. The methodology adhered to the guidelines outlined in the Cochrane Handbook for Umbrella Reviews of Systematic Reviews [22–24]. In the reporting, we followed the PRISMA statement and the AMSTAR 2 guidelines (**Table 1**) [36,37]. Two authors were responsible for the processes of literature search, study selection, data extraction, and quality assessment. Any disagreements encountered during these processes were resolved through discussion or, if necessary, by consultation with the third reviewer to reach a consensus [38].

**Table 1:** Methodological quality appraisal of the included systematic reviews and meta-analyses using AMSTAR 2*

Study	Rating categories																Overall rating
	1	2	3	4	5	6	7	8	9	10	11	12	13	14	15	16	
Sheng *et al.*, 2014 [25]	Y	Y	Y	Y	Y	Y	Y	Y	Y	Y	Y	N	Y	Y	Y	Y	Moderate
Chung *et al.*, 2017 [26]	Y	N	Y	Y	Y	Y	Y	Y	Y	Y	Y	Y	Y	N	Y	Y	Low
Young *et al.*, 2018 [27]	Y	Y	Y	Y	Y	Y	Y	Y	Y	Y	Y	Y	Y	Y	Y	Y	High
Bogataj *et al.*, 2020 [28]	Y	Y	Y	Y	Y	Y	Y	Y	Y	Y	Y	Y	Y	Y	N	Y	Low
Ferreira *et al.*, 2019 [29]	Y	Y	Y	Y	Y	Y	Y	Y	Y	Y	N	Y	Y	N	Y	Y	Low
Huang *et al.*, 2019 [30]	Y	Y	Y	Y	Y	Y	Y	Y	Y	Y	Y	Y	Y	Y	Y	Y	High
Kirkman *et al.*, 2019 [31]	Y	Y	Y	Y	Y	Y	Y	Y	Y	Y	N	Y	Y	N	Y	Y	Low
Molsted *et al.*, 2019 [32]	Y	Y	Y	Y	Y	Y	Y	Y	Y	Y	Y	N	Y	Y	Y	Y	Moderate
Salhab *et al.*, 2019 [33]	Y	Y	Y	Y	Y	Y	Y	Y	Y	Y	Y	N	Y	N	Y	Y	Moderate
Ferrari *et al.*, 2020 [34]	Y	Y	Y	Y	Y	Y	Y	Y	Y	Y	Y	Y	Y	N	Y	Y	Moderate
Yu *et al.*, 2024 [35]	Y	Y	Y	Y	Y	Y	Y	Y	Y	Y	Y	Y	Y	Y	Y	Y	High

N – no, Y – yes

*Items 1–16 represent the methodological domains assessed using AMSTAR 2. Q1: Did the research questions and inclusion criteria for the review include the components of PICO?, Q2: Did the report of the review contain an explicit statement that the review methods were established prior to the conduct of the review, and did the report justify any significant deviations from the protocol?, Q3: Did the review authors explain their selection of the study designs for inclusion in the review?, Q4: Did the review authors use a comprehensive literature search strategy?, Q5: Did the review authors perform study selection in duplicate?, Q6: Did the review authors perform data extraction in duplicate?, Q7: Did the review authors provide a list of excluded studies and justify the exclusions?, Q8: Did the review authors describe the included studies in adequate detail?, Q9: Did the review authors use a satisfactory technique for assessing the risk of bias (RoB) in individual studies that were included in the review?, Q10: Did the review authors report on the sources of funding for the studies included in the review?, Q11: If meta-analysis was performed, did the review authors use appropriate methods for statistical combination of results?, Q12: If meta-analysis was performed, did the review authors assess the potential impact of RoB in individual studies on the results of the meta-analysis or other evidence synthesis?, Q13: Did the review authors account for RoB in primary studies when interpreting/discussing the results of the review?, Q14: Did the review authors provide a satisfactory explanation for, and discussion of, any heterogeneity observed in the results of the review?, Q15: If they performed quantitative synthesis, did the review authors carry out an adequate investigation of publication bias (small study bias) and discuss its likely impact on the results of the review?, Q16: Did the review authors report any potential sources of conflict of interest, including any funding they received for conducting the review? The overall rating represents the level of confidence in the results of each review and was classified as high, moderate, low, or critically low

### Search methodology

We systematically searched Embase, PubMed/MEDLINE, the Cochrane Library, and Web of Science through January 2026. We designed the search strategy using a combination of subject terms and free words related to the core concepts. Key search terms included ‘exercise therapy’, ‘haemodialysis’, ‘renal insufficiency’, ‘chronic kidney disease’, ‘end-stage renal disease’, ‘meta-analysis’, and ‘systematic review’ (Text S1 in the **Online Supplementary Document**).

### Study selection process

In the study selection process, we followed the Population, Intervention, Comparison, Outcomes, and Study type framework. The population inclusion criteria were: adult patients (aged ≥18 years) diagnosed with chronic kidney disease, primarily at the ESRD stage, who were undergoing maintenance haemodialysis. Intervention inclusion criteria were: intradialytic or non-intradialytic exercise interventions, including aerobic training, resistance training, or a combination of both. Comparison inclusion criteria were: control groups receiving usual haemodialysis care, no exercise, sham exercise, or low-intensity activities. Outcomes inclusion criteria were: reporting on at least one of the following outcomes – Kt/V, VO_2_ peak, functional capacity (six-minute walk test (6MWT)), health-related quality of life (physical component score (PCS), and mental component score (MCS) of the short-form 36 health questionnaire), systolic blood pressure (SBP) and diastolic blood pressure (DBP), CRP, depression scores, or serum phosphorus levels. Study type inclusion criteria referred to systematic reviews or meta-analyses of RCTs.

We excluded narrative reviews, letters, editorials, conference abstracts, study protocols, and network meta-analyses. Further, we excluded meta-analyses for which extractable data on intradialytic exercise outcomes could not be obtained, and studies for which the full text was unavailable. We also excluded studies that were not in English. Reviews focusing on interventions that were not relevant to the defined exercise modalities were excluded. If multiple meta-analyses from the same author group used overlapping data sets, the most recent or most comprehensive publication was selected to avoid duplication (Table S1 in the **Online Supplementary Document**).

### Overlapping discovery and processing

If two or more meta-analyses evaluated the same outcome, the original studies they included could overlap. To quantify the extent of overlap for each outcome, we calculated the corrected covered area (CCA) using the GROOVE methodology [39]. For each outcome, we constructed a study-by-review matrix to identify all unique primary studies (r) and the total number of inclusions across the relevant meta-analyses (N). The CCA was derived as:



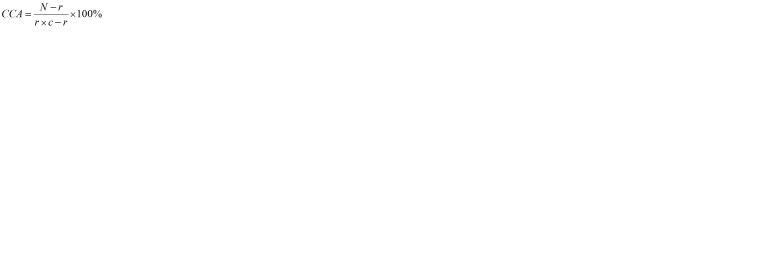



where ‘c’ represents the number of meta-analyses contributing to that outcome. We classified the overlap as slight (<5%), moderate (5% to <10%), high (10% to <15%), or very high (≥15%). Two reviewers independently conducted the overlap assessment using GROOVE. Any discrepancies in the interpretation of overlap were resolved by consulting a third reviewer. To manage overlapping data and minimise bias in the synthesis, we applied the following hierarchical decision rules. If there was overlap between Cochrane and non-Cochrane reviews, we prioritised results from the Cochrane review. For a high or very high degree of overlap among non-Cochrane reviews, precedence was given to the meta-analysis with the highest AMSTAR 2 quality score. If AMSTAR 2 scores were identical, the meta-analysis with the largest number of included RCTs or the most recent publication date was selected. For slight or moderate overlap, we considered and included results from all relevant meta-analyses in the descriptive summary (Figure S1 in the **Online Supplementary Document**).

### Data extraction and quality assessment

We assessed inter-rater agreement for study selection and data extraction using Cohen’s κ. The κ value was 0.87 (95% confidence interval (CI) = 0.82, 0.92), indicating almost perfect agreement. We used a standardised data extraction form to collect the following information from each included meta-analysis: first author, publication year, journal discipline, date of the last search, number and design of included primary studies, total sample size, characteristics of the participant population, details of the exercise intervention and comparator, and all reported outcome measures with their respective effect sizes, CIs, *P*-values, and *I*^2^ statistic [40]. We used the GRADE approach to rate the overall certainty of evidence for each outcome, considering the entire body of evidence from all relevant meta-analyses [41]. For each outcome, two reviewers independently assessed the following domains: risk of bias, inconsistency, indirectness, imprecision, and publication bias. Each outcome was rated as having high, moderate, low, or very low certainty. Additionally, we classified the resulting evidence into four categories based on evidence classification criteria – convincing evidence (level one), highly suggestive evidence (level two), suggestive evidence (level three), weak evidence (level four), and not significant (**Table 2**; Table S2 in the **Online Supplementary Document**).

**Table 2:** Evidence classification criteria

Evidence class	Criteria
I	>1000 cases (or >20,000 participants for continuous outcomes); statistical significance at *P* < 10^−6^ (random effects); no evidence of small study effects and excess significance bias; 95% prediction interval excluded null value; no large heterogeneity (*I*^2^<50)
II	>1000 cases (or >20,000 participants for continuous outcomes); statistical significance at *P* < 10^−6^ (random effects); the largest study with 95% CI excluding null value
III	>1000 cases (or >20,000 participants for continuous outcomes) and statistical significance at *P* < 0.001
IV	Remaining significant associations with *P* < 0.05
Non-significant	*P* > 0.05

CI – confidence interval

### Data synthesis

We extracted effect estimates including mean difference (MD), weighted mean difference (WMD), standardised mean difference (SMD), 95% CIs, *P*-values, and *I*^2^ statistics. The *I*^2^ values were interpreted as follows: <25% low heterogeneity; 25–50% low to moderate heterogeneity; 50–75% moderate to substantial heterogeneity; and >75% substantial heterogeneity. A *P*-value of <0.05 for the Q-test was considered indicative of significant statistical heterogeneity. We explored significant clinical heterogeneity through subgroup analyses and, where appropriate, sensitivity analyses. Regarding the statistical model for pooling data, we used a fixed-effect model when heterogeneity was low (*I*^2^ < 25 and *P* ≥ 0.10), and a random-effects model (DerSimonian-Laird) when heterogeneity was moderate to substantial (*I*^2^ ≥ 25 and *P* < 0.10), as this accounts for variability across studies.

## RESULTS

### Search results

**Figure 1:**
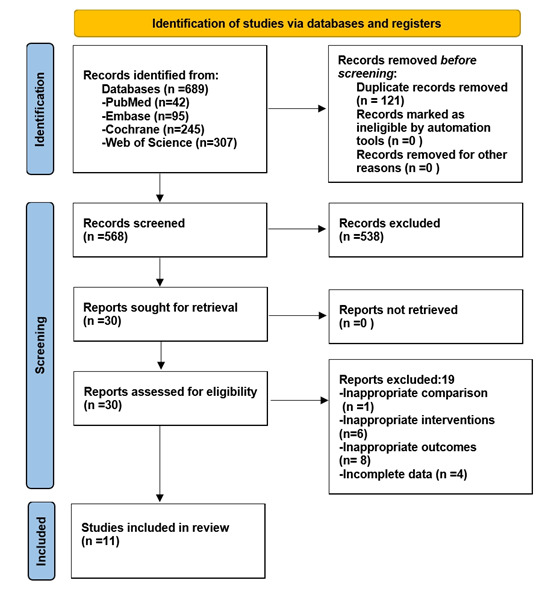
PRISMA flow diagram to show study selection.

The initial search included 689 systematic reviews. After removing 121 duplicates, we screened 568 records. Of these, we excluded 538 based on the title and abstract, leaving 30 full-text reports for eligibility assessment. A further 19 were excluded through reading the full text, resulting in 11 meta-analyses included in the final analysis (**Figure 1**).

### Study characteristics

The included meta-analyses were published between 2014 and 2024 and exclusively enrolled adult patients aged >18 years. All reviews reported sample sizes [25–35], with only three exceeding 1000 participants [32,34,35]. Regarding the evidence classification of the extracted outcomes, 23 were assigned to level four, 10 to level three, and 21 were considered non-significant. According to GRADE, six outcomes were rated as moderate certainty, seven as very low, and 41 as low certainty. AMSTAR 2 appraisals revealed high methodological quality in three meta-analyses and moderate or low in the remaining four. The calculated CCA values ranged from 15.4–33.3% across outcomes, all falling into the very high overlap category. Given this substantial overlap, simply pooling all available meta-analyses would lead to severe double-counting of primary data and overestimated precision. We therefore applied pre-specified hierarchical selection rules (Cochrane priority, AMSTAR 2 rating, number of RCTs, and recency) to retain the most appropriate single meta-analysis per outcome-modality combination for the primary synthesis (**Table 3**).

**Table 3:** Basic information of the included studies

Study	Region	Treatment	Control	Duration of exercise	Dialysis frequency	Studies included, n	Sample size	Age in years	Female, %	Last search date	Outcomes
Sheng *et al.*, 2014 [25]	China	AT, RT	No exercise	8–40 weeks	3 times a week	24	997	53.90	37%	February 2014	Kt/V, VO_2_ peak, SF-36, and adverse events
Chung *et al.*, 2017 [26]	Taiwan	AT, RT	No exercise	8 weeks	10–60 min, 3 times a week	17	651	NA	NA	January 2015	6MWT, VO_2_ peak, haemoglobin, albumin, depression, PCS, MCS, and adverse events
Young *et al.*, 2018 [27]	UK	CT	No exercise	14 weeks (6–26 weeks)	31 minutes (range 20–45), twice a week	8	369	x̄ (SD) = 53.6 (11.1)	44%	28 February 2018	VO_2_ peak, 6MWT, PCS, MCS, SBP, DBP, pulse wave velocity, and physical function
Bogataj *et al.*, 2020 [28]	Slovenia	AT, RT, CT	Pre-post intervention	12 weeks (8–40 weeks)	13–90 min, 3 times a week	33	1274	x̄ (SD) = 55.7 (9.6)	NA	March 2019	6MWT, Oxygen consumption, CRP, and10-repetition sit-to-stand test
Ferreira *et al.*, 2019 [29]	Brazil	RT	No exercise	6–25 weeks	NA	23	NA	NA	NA	July 2018	Creatinine, phosphate, potassium, and Kt/V
Huang *et al.*, 2019 [30]	China	AT, RT, CT	Usual care or sham activity	12 weeks (8–48 weeks)	15–90 min, 3 times a week	20	677	x̄ (SD) = 52.0 (12.5)	39	November 2018	Kt/V, SBP, DBP, VO_2_ peak, 6MWT, PCS, MCS, and SF-36
Kirkman *et al.*, 2019 [31]	UK	AT, RT	No exercise	8 weeks	60 min, 2–3 times a week	15	307	18–76	NA	November 2018	Kt/V, urea, creatinine, beta-2-microglobulin, phosphate, and potassium
Molsted *et al.*, 2019 [32]	NA	AT, RT, CT	Usual care or sham exercises	NA	NA	8	290	x̄ (SD) = 52.1 (13.2)	39	October 2017	Muscle size, 1-5RM tests, dynamometers, 6MWT, SF36, PCS, MCS, kidney disease questionnaire, and adverse effects
Salhab *et al.*, 2019 [33]	Netherlands	AT	Usual care	8–24 weeks	15–60 min, 2–3 times a week	22	706	43.3–72.5 years	40	June 2018	PCS, MCS, serum phosphorus, dialysis efficiency, inflammatory status, vitamin D3, parathyroid hormone, intake of phosphate binders, mortality, and hospitalisation rate
Ferrari *et al.*, 2020 [34]	Brazil	AT, RT, CT	Usual care or sham exercises	48 months	3 times a week	50	1,757	20–73.9	NA	July 2019	Kt/V, SBP, DBP, VO_2_ peak, 6MWT, haemoglobin, CRP, IL-6, andTC
Yu *et al.*, 2024 [35]	China	AT, RT, CT	Usual care, sham exercise, and no exercise	8–48 weeks	20–90 minutes, 3 times a week	22	1,059	x̄ (SD) = 53.0 (10.4)	40	April 2023	Beck depression inventory, self-rating depression scale, hospital anxiety, and depressive depression scale

AT – aerobic training, CRP –C-reactive protein, CT – combined training, DBP – diastolic blood pressure, IL-6 – Interleukin-6, Kt/V – urea clearance index, MCS – mental component scores, NA – not available, PCS — physical component scores, RM – repetition maximum, RT – resistance training, SBP – systolic blood pressure, SD – standard deviation, SF-36 – short-form 36 health questionnaire, TC – total cholesterol, VO_2_ peak – peak oxygen consumption, 6MWT – six-minute walk test, x̄ – mean

### Results of meta-analyses of functional scores

#### Dialysis adequacy

Regarding dialysis adequacy, Kt/V was reported in five meta-analyses [25,29,30,33,34]. After overlap management, the analysis by Ferrari *et al.* [34] was selected for aerobic and resistance exercise because it had the highest AMSTAR 2 score and the largest number of included studies (n = 50) among all meta-analyses reporting these modalities. For combined exercise, Huang *et al.* [30] was chosen as it was the only meta-analysis that specifically analysed combined training for Kt/V and had low heterogeneity (*I*^2^ = 0). Aerobic exercise significantly improved Kt/V (WMD = 0.08; 95% CI = 0.00, 0.15, *P* = 0.04, *I*^2^ = 56). Resistance exercise showed no significant effect (WMD = 0.10; 95% CI = −0.01, 0.20, *P* = 0.06, *I*^2^ = 0). Combined exercise also did not significantly affect Kt/V (SMD = 0.19; 95% CI = –0.06, 0.43, *P* = 0.14, *I*^2^ = 0).

#### Cardiorespiratory fitness

Cardiorespiratory fitness and VO_2_ peak were assessed in six meta-analyses [25–28,30,34]. Based on the highest AMSTAR 2 score and largest sample size, Ferrari *et al.* [34] was considered the best available evidence for both aerobic and combined exercise. Both aerobic (WMD = 2.07; 95% CI = 0.42, 3.72, *P* = 0.01, *I*^2^ = 0) and combined exercise (WMD = 5.41; 95% CI = 4.03, 6.79, *P* < 0.00001, *I*^2^ = 0%) significantly increased VO_2_ peak. No resistance-only meta-analysis reported this outcome.

#### Functional capacity

The 6MWT was assessed in six meta-analyses [25–28,30,34]. Following the same hierarchical approach, Ferrari *et al.* [34] was selected for all three modalities because it had the highest methodological quality (high AMSTAR 2), the largest number of primary studies, and the most recent search date. Aerobic training (WMD = 64.98; 95% CI = 43.96, 86.11, *P* < 0.00001, *I*^2^ = 0) and resistance training (WMD = 68.50; 95% CI = 29.05, 107.96, *P* = 0.0007, *I*^2^ = 36%) significantly improved 6MWT, whereas combined training did not (WMD = 36.37; 95% CI = –13.73, 86.46, *P* = 0.15, *I*^2^ = 0).

#### Blood pressure

Blood pressure (SBP and DBP) was reported in four meta-analyses [25,27,30,34]. Ferrari *et al.* [34] provided the best evidence for both aerobic and combined exercise due to its high AMSTAR 2 rating and comprehensive data extraction. Aerobic exercise significantly reduced SBP (WMD = −10.07; 95% CI = −16.35, −3.78, *P* = 0.002, *I*^2^ = 44) but not DBP (WMD = −2.96; 95% CI = −7.71, 1.78, *P* = 0.22, *I*^2^ = 65). Conversely, combined exercise significantly lowered DBP (WMD = −5.76; 95% CI = −8.83, −2.70, *P* = 0.0002, *I*^2^ = 0%) but not SBP (WMD = −4.33; 95% CI = −9.75, 1.08, *P* = 0.12, *I*^2^ = 0). No resistance-only meta-analyses reported blood pressure outcomes.

#### Physical and mental component scores

In the six meta-analyses, PCS and MCS were examined [25–27,30,32,33]. For aerobic exercise, Young *et al.* [27] was selected because it was the only meta-analysis with low heterogeneity and a specific focus on intradialytic cycling, which best represented the aerobic intervention. Aerobic exercise did not significantly improve PCS (MD = 1.97; 95% CI = –8.27, 12.22, *P* = 0.71, *I*^2^ = 18%) or MCS (MD = 3.37; 95% CI = −7.94, 14.68, *P* = 0.56). For resistance exercise, Molsted *et al.* [32] was the only meta-analysis reporting PCS, showing a significant improvement (MD = 10.05; 95% CI = 2.95, 17.14, *P* = 0.0006, *I*^2^ = 0%). For combined exercise, Huang *et al.* [30] was selected as the most comprehensive meta-analysis with low to moderate heterogeneity, demonstrating significant improvements in both PCS (SMD = 0.34; 95% CI = 0.09, 0.59, *P* = 0.007, *I*^2^ = 27) and MCS (SMD = 0.27; 95% CI = 0.02, 0.51, *P* = 0.03, *I*^2^ = 0%).

#### Inflammation

In two meta-analyses, CRP was examined [28,34]. For aerobic and resistance exercise, Ferrari *et al.* [34] was prioritised because of its high AMSTAR 2 quality and very low heterogeneity (*I*^2^ = 0% for aerobic, 10% for resistance). Aerobic exercise significantly reduced CRP (WMD = −3.28; 95% CI = −4.68, −1.88, *P* < 0.00001, *I*^2^ = 0), whereas resistance exercise did not (WMD = −0.50; 95% CI = −1.52, 0.52, *P* = 0.34, *I*^2^ = 10). For combined exercise, Bogataj *et al.* [28] provided the only available estimate, showing a significant reduction (SMD = −0.82; 95% CI = −1.04, −0.60, *P* < 0.001).

#### Depression

Depressive symptoms were evaluated in two meta-analyses [26,35]. After overlap management, Yu *et al.* [35] was selected as the best evidence for all three exercise modalities because it was the most recent, included the largest number of RCTs (n = 22), and had substantially lower heterogeneity for aerobic and resistance comparisons compared with the older meta-analyses. Aerobic exercise (SMD = −0.93; 95% CI = −1.32, −0.55, *P* < 0.001, *I*^2^ = 0) and combined exercise (SMD = −0.85; 95% CI = −1.29, −0.41, *P* < 0.001, *I*^2^ = 76.07) significantly reduced depression scores, while resistance exercise showed no significant effect (SMD = −0.40; 95% CI = −0.96, 0.17, *P* = 1.00, *I*^2^ = 0).

#### Serum phosphorus

Serum phosphorus was examined in two meta-analyses [29,33]. Ferreira *et al.* [29] was chosen as the best evidence for aerobic exercise because it provided an MD with a full 95% CIs, whereas the other meta-analysis reported only a vague non-significant statement. Aerobic exercise did not significantly affect serum phosphorus (MD = −1.06; 95% CI = −2.72, 0.60, *P* = 0.21, *I*^2^ = 98). No resistance or combined training data were available (Table S3 in the **Online Supplementary Document**).

## DISCUSSION

This umbrella review provides a comprehensive and methodologically rigorous synthesis of 11 meta-analyses investigating the effects of exercise in patients undergoing haemodialysis. The findings indicate that aerobic, resistance, and combined exercise interventions confer distinct benefits across a range of clinical, functional, and patient-reported outcomes [25–35].

Aerobic exercise significantly improved Kt/V, VO_2_ peak, functional capacity (6MWT), SBP, CRP, and depressive symptoms. These results corroborate previous studies showing that aerobic training enhances cardiovascular function, microcirculation, and solute clearance during dialysis [42,43]. Andrade *et al.* [44] demonstrated that patients’ cardiorespiratory function was significantly improved by aerobic exercise. Although aerobic exercise has been associated with reductions in inflammatory markers such as CRP in several studies [45,46], this finding is not universal; other trials report no significant effect [47–49]. This discrepancy may be attributable to differences in exercise intensity, duration, or patient characteristics. Liu *et al.* [50] demonstrated that aerobic exercise may reduce depression levels in patients. However, the improvements in DBP, PCS, and MCS with aerobic exercise were not significant, likely due to the short intervention duration or to exercise intensity not reaching thresholds sufficient to affect these indicators. Improvement in PCS and MCS by aerobic exercise alone would also be insufficient to modify these parameters.

Resistance exercise produced robust improvements in functional capacity (6MWT) and the physical component of quality of life, yet did not influence Kt/V, CRP, or depression. Improvements in patients’ lower-extremity strength, endurance, and ability to perform activities of daily living suggest that the core benefits of resistance exercise primarily enhance muscle function rather than modulate systemic metabolism or inflammation. The improvement in PCS is in line with earlier findings. The study by Perez-Dominguez [51] similarly showed positive results in 6MWT and PCS without significant improvement in depression. However, in contrast to the present findings, a study by Deus *et al.* [52] showed that resistance exercise significantly improved quality of life, antioxidant capacity, and depression in dialysis patients. This should be interpreted with caution. The quality of life and emotional state of dialysis patients are generally low and are influenced by factors such as age, gender, functional status, and work status.

Combined aerobic and resistance training demonstrated the broadest range of benefits, significantly improving VO_2_ peak, DBP, both physical and mental quality of life, CRP, and depression. Nevertheless, there were no significant differences in urea clearance, 6MWT, or SBP. Basir and Mirzaei [53] showed improvements in cardiorespiratory fitness. Frih *et al.* [54] demonstrated improvements in physical and mental health, as well as in depression levels, among dialysis patients through combined exercise. Meléndez Oliva showed that combining exercise significantly improved inflammation levels [15]. Our results show that aerobic exercise was effective only in reducing systolic blood pressure, not diastolic blood pressure; the opposite was true for combined exercise. This may be due to aerobic exercise improving the compliance of large arteries but not reversing the short-term inward remodelling of small arteries, and to the reflex activation of sympathetic nerve fibres after exercise, maintaining DBP. In contrast, combined exercise counteracts the SBP-lowering effect of aerobic exercise because resistance training can elevate blood pressure through several mechanisms.

The clinical significance of some statistically significant findings warrants careful consideration. For Kt/V, the improvement with aerobic exercise (WMD = 0.08; 95% CI = 0.00l, 0.15) represents a modest absolute change. According to the Kidney Disease Outcomes Quality Initiative guidelines, a Kt/V≥1.2 is considered the minimum target for adequate dialysis [55]. An increase of 0.08 is therefore relatively small and may not be sufficient to move a patient from the insufficient to the adequate range in most cases. While statistically significant, this effect may not translate into clinically meaningful improvements in dialysis adequacy. In contrast, the improvements in the 6MWT (64.98 m for aerobic exercise, 68.50 m for resistance exercise) substantially exceed the minimal clinically important difference of 14–15 m established for exercise capacity in this population [56], suggesting clinically meaningful functional gains.

We conducted an umbrella review in accordance with established methodological guidance for overviews of systematic reviews. To ensure transparency and reproducibility, we appraised the quality of included meta-analyses using AMSTAR 2, rated outcome-specific certainty with GRADE, and quantified primary study overlap *via* the GROOVE tool. Despite this rigorous framework, several outcomes showed considerable statistical heterogeneity. For instance, the *I*^2^ value for Kt/V following aerobic exercise was 56%, and for depression after combined training it reached 76%. Such heterogeneity is not unexpected in this field and likely stems from multiple sources, including differences in exercise protocols such as frequency, intensity, session duration, and timing relative to dialysis sessions; variability in patient characteristics like age, comorbidities, and baseline functional status; diverse control conditions ranging from usual care to sham exercise or low-intensity activity; inconsistent outcome measurement tools and follow-up durations; and variations in dialysis prescriptions, for example low-flux *vs.* high-flux membranes or blood flow rates. This degree of heterogeneity limits the precision of our pooled estimates and reinforces the need for more standardised intervention protocols.

Several limitations warrant caution when interpreting our findings. The included meta-analyses may carry inherent biases; four were rated as having low methodological quality on AMSTAR 2. Furthermore, despite our use of the GROOVE tool to select the most representative meta-analysis for each outcome, primary study overlap remained substantial, potentially leading to overly precise estimates. It is also important to note that no outcome reached high GRADE certainty; 41 of 54 outcomes (76%) were rated low certainty, and seven were rated very low. Thus, although the direction of effect was consistently favourable for many outcomes, the confidence in these effect estimates is limited. Only three of the eleven included meta-analyses had sample sizes exceeding 1000 participants, meaning that most evidence derives from relatively small meta-analyses – potentially underpowered to detect modest but clinically important effects and susceptible to small-study bias. In addition, most primary RCTs lacked long-term follow-up (beyond 12 months) and used heterogeneous outcome definitions, limiting comparability across studies. Patient characteristics – such as comorbidities, concurrent medications, and baseline physical function – were often inadequately reported or controlled for in the original research. Our restriction to English-language publications may also have introduced language bias. For most outcomes, the number of available meta-analyses was too small to formally assess publication bias. Lastly, we did not perform a *de novo* meta-analysis of primary studies, which could have enabled more consistent data handling across studies. Taken together, these limitations argue for a cautious interpretation and cautious generalisation of our findings.

In light of the available evidence, structured exercise programs should be incorporated into the routine management of haemodialysis patients. Clinically, exercise prescriptions should be tailored to individual patient needs: aerobic exercise to improve cardiopulmonary function and dialysis efficiency; resistance training to enhance muscle strength and physical function; and combined exercise for those seeking comprehensive rehabilitation. At the policy level, implementation should be facilitated by integrating exercise professionals and equipment into dialysis units, along with insurance coverage, to improve accessibility and long-term adherence.

Future research should focus on conducting large-scale, long-term RCTs to examine the effects of exercise on hard endpoints such as hospitalisation rates and mortality. Furthermore, reporting methods for exercise parameters require standardisation to clarify optimal intensity, frequency, and duration. The biological mechanisms underpinning exercise benefits should be elucidated, including their effects on inflammatory pathways, vascular function, and neuroendocrine regulation. Research populations should be broadened to include non-English-speaking regions and patients with diverse comorbidities, thereby strengthening the generalizability of the findings. Finally, future research may develop artificial intelligence models to provide haemodialysis patients with more accessible and personalised exercise intervention programs.

## CONCLUSIONS

In patients undergoing haemodialysis, aerobic exercise may improve cardiorespiratory fitness, dialysis efficiency, inflammation, and depression; resistance exercise may enhance muscle strength and physical function; and combined aerobic-resistance training may offer the broadest biopsychosocial benefits. These findings support the consideration of individualised exercise prescriptions, but high-certainty evidence is lacking. Future research should prioritise long-term trials and develop personalised exercise regimens using digital health technologies.

## Additional material


Online Supplementary Document

## Data Availability

**Data availability:** All data generated or analysed during this study are fully available within the article and its supplementary materials.

## References

[1] Kovesdy CP Epidemiology of chronic kidney disease: an update 2022. Kidney Int Suppl (2011). 2022;12:7–11. 10.1016/j.kisu.2021.11.003 35529086 PMC9073222

[2] Jager KJ Kovesdy C Langham R Rosenberg M Jha V Zoccali C A single number for advocacy and communication-worldwide more than 850 million individuals have kidney diseases. Nephrol Dial Transplant. 2019;34:1803–5. 10.1093/ndt/gfz174 31566230

[3] Foreman KJ Marquez N Dolgert A Fukutaki K Fullman N McGaughey M Forecasting life expectancy, years of life lost, and all-cause and cause-specific mortality for 250 causes of death: reference and alternative scenarios for 2016–40 for 195 countries and territories. Lancet. 2018;392:2052–90. 10.1016/S0140-6736(18)31694-5 30340847 PMC6227505

[4] Rout P, Aslam A. End-Stage Renal Disease. Treasure Island, Florida, USA: StatPearls; 2025. 29763036

[5] Saran R Robinson B Abbott KC Agodoa LY Bhave N Bragg-Gresham J US renal data system 2017 annual data report: epidemiology of kidney disease in the United States. Am J Kidney Dis. 2018;71:A7. 10.1053/j.ajkd.2018.01.002 29477157 PMC6593155

[6] Elshahat S Cockwell P Maxwell AP Griffin M O’Brien T O’Neill C The impact of chronic kidney disease on developed countries from a health economics perspective: A systematic scoping review. PLoS One. 2020;15:e0230512. 10.1371/journal.pone.0230512 32208435 PMC7092970

[7] P T Quality of life of patients undergoing hemodialysis. Asian J Pharm Clin Res. 2018;11:219–23. 10.22159/ajpcr.2018.v11i4.24007

[8] Joshi VD Quality of life in end stage renal disease patients. World J Nephrol. 2014;3:308–16. 10.5527/wjn.v3.i4.308 25374827 PMC4220366

[9] Musolino M Presta P Cianfrone P Errante MA Andreucci M Coppolino G Self-Reported Physical Inactivity and Mood Disturbances in End-Stage Kidney Disease (ESKD) Patients on Chronic Dialysis Treatment. J Clin Med. 2023;12:7160. 10.3390/jcm12227160 38002771 PMC10672008

[10] Cheng TC Huang SH Kao CL Hsu PC Muscle Wasting in Chronic Kidney Disease: Mechanism and Clinical Implications-A Narrative Review. Int J Mol Sci. 2022;23:6047. 10.3390/ijms23116047 35682722 PMC9181340

[11] Dziubek W Bulińska K Kusztal M Kowalska J Rogowski Ł Zembroń-Łacny A Evaluation of exercise tolerance in dialysis patients performing tai chi training: preliminary study. Evid Based Complement Alternat Med. 2016;2016:5672580. 10.1155/2016/5672580 27547228 PMC4980525

[12] Bakker EA Zoccali C Dekker FW Eijsvogels TM Jager KJ Assessing physical activity and function in patients with chronic kidney disease: a narrative review. Clin Kidney J. 202;14:768–79. 10.1093/ckj/sfaa156 33777360 PMC7986327

[13] Wathanavasin W Banjongjit A Avihingsanon Y Praditpornsilpa K Tungsanga K Eiam-Ong S Prevalence of Sarcopenia and Its Impact on Cardiovascular Events and Mortality among Dialysis Patients: A Systematic Review and Meta-Analysis. Nutrients. 2022;14:4077. 10.3390/nu14194077 36235729 PMC9572026

[14] Yamagata K Hoshino J Sugiyama H Hanafusa N Shibagaki Y Komatsu Y Clinical practice guideline for renal rehabilitation: systematic reviews and recommendations of exercise therapies in patients with kidney diseases. Ren Replace Ther. 2019;5:28. 10.1186/s41100-019-0209-8

[15] Meléndez Oliva E Villafañe JH Alonso Pérez JL Alonso Sal A Molinero Carlier G Quevedo García A Effect of Exercise on Inflammation in Hemodialysis Patients: A Systematic Review. J Pers Med. 2022;12:1188. 10.3390/jpm12071188 35887685 PMC9322638

[16] Rohmah SN Puspitasari M Prasanto H Wardhani Y Kuswadi I Dhamarjati A Effect of intradialytic aerobic exercise intervention on dialysis adequacy and quality of life in patients with end-stage kidney disease undergoing hemodialysis at Dr. Sardjito General Hospital, Indonesia. Int Urol Nephrol. 2024;56:3595–604. 10.1007/s11255-024-04100-x 38850394

[17] Matsuzawa R Hoshi K Yoneki K Harada M Watanabe T Shimoda T Exercise training in elderly people undergoing hemodialysis: a systematic review and meta-analysis. Kidney Int Rep. 2017;2:1096–110. 10.1016/j.ekir.2017.06.008 29270518 PMC5733833

[18] Manfredini F Mallamaci F D’Arrigo G Baggetta R Bolignano D Torino C Exercise in patients on dialysis: a multicenter, randomized clinical trial. J Am Soc Nephrol. 2017;28:1259–68. 10.1681/ASN.2016030378 27909047 PMC5373448

[19] M Abd E-Kader S Refaey N AlKhateeb AM AlFawaz SS Neamatallah ZA Alabasi UM Exercise tolerance and fatigue response to aerobic versus resisted exercise among hemodialysis patients. Afr Health Sci. 2024;24:420–6. 10.4314/ahs.v24i2.4240190536

[20] Bakaloudi DR Siargkas A Poulia KA Dounousi E Chourdakis M The Effect of Exercise on Nutritional Status and Body Composition in Hemodialysis: A Systematic Review. Nutrients. 2020;12:3071. 10.3390/nu12103071 33050111 PMC7601723

[21] Ioannidis JP Integration of evidence from multiple meta-analyses: a primer on umbrella reviews, treatment networks and multiple treatments meta-analyses. CMAJ. 2009;181:488–93. 10.1503/cmaj.081086 19654195 PMC2761440

[22] Blom AW Donovan RL Beswick AD Whitehouse MR Kunutsor SK Common elective orthopaedic procedures and their clinical effectiveness: umbrella review of level 1 evidence. BMJ. 2021;374. 10.1136/bmj.n1511 34233885 PMC8262448

[23] Smith V Devane D Begley CM Clarke M Methodology in conducting a systematic review of systematic reviews of healthcare interventions. BMC Med Res Methodol. 2011;11:15. 10.1186/1471-2288-11-15 21291558 PMC3039637

[24] Fusar-Poli P Radua J Ten simple rules for conducting umbrella reviews. Evid Based Ment Health. 2018;21:95–100. 10.1136/ebmental-2018-300014 30006442 PMC10270421

[25] Sheng K Zhang P Chen L Cheng J Wu C Chen J Intradialytic Exercise in Hemodialysis Patients: A Systematic Review and Meta-Analysis. Am J Nephrol. 2014;40:478–90. 10.1159/000368722 25504020

[26] Chung YC Yeh ML Liu YM Effects of intradialytic exercise on the physical function, depression and quality of life for haemodialysis patients: a systematic review and meta-analysis of randomised controlled trials. J Clin Nurs. 2017;26:1801–13. 10.1111/jocn.13514 27532211

[27] Young HML March DS Graham-Brown MPM Jones AW Curtis F Grantham CS Effects of intradialytic cycling exercise on exercise capacity, quality of life, physical function and cardiovascular measures in adult haemodialysis patients: a systematic review and meta-analysis. Nephrol Dial Transplant. 2018;33:1436–45. 10.1093/ndt/gfy045 29608708

[28] Bogataj Š Pajek M Pajek J Ponikvar JB Paravlic A Exercise-based interventions in hemodialysis patients: A systematic review with a meta-analysis of randomized controlled trials. J Clin Med. 2019;9:43. 10.3390/jcm9010043 31878176 PMC7019709

[29] Ferreira GD Bohlke M Correa CM Dias EC Orcy RB Does Intradialytic Exercise Improve Removal of Solutes by Hemodialysis? A Systematic Review and Meta-analysis. Arch Phys Med Rehabil. 2019;100:2371–80. 10.1016/j.apmr.2019.02.009 30922880

[30] Huang M Lv A Wang J Xu N Ma G Zhai Z Exercise Training and Outcomes in Hemodialysis Patients: Systematic Review and Meta-Analysis. Am J Nephrol. 2019;50:240–54. 10.1159/000502447 31454822

[31] Kirkman DL Scott M Kidd J Macdonald JH The effects of intradialytic exercise on hemodialysis adequacy: A systematic review. Semin Dial. 2019;32:368–78. 10.1111/sdi.12785 30968465

[32] Molsted S Bjørkman ASD Lundstrøm LH Effects of strength training to patients undergoing dialysis: A systematic review. Dan Med J. 2019;66:A5526. 30573007

[33] Salhab N Karavetian M Kooman J Fiaccadori E El Khoury CF Effects of intradialytic aerobic exercise on hemodialysis patients: a systematic review and meta-analysis. J Nephrol. 2019;32:549–66. 10.1007/s40620-018-00565-z 30659520 PMC6588711

[34] Ferrari F Helal L Dipp T Soares D Soldatelli Â Mills AL Intradialytic training in patients with end-stage renal disease: a systematic review and meta-analysis of randomized clinical trials assessing the effects of five different training interventions. J Nephrol. 2020;33:251–66. 10.1007/s40620-019-00687-y 31865607

[35] Yu H Huang M Tao Y Li S Wang J Li P The effects of exercise training interventions on depression in hemodialysis patients. Front Psychiatry. 2024;14:1321413. 10.3389/fpsyt.2023.1321413 38260806 PMC10800967

[36] Page MJ McKenzie JE Bossuyt PM Boutron I Hoffmann TC Mulrow CD The PRISMA 2020 statement: An updated guideline for reporting systematic reviews. Int J Surg. 2021;88:105906. 10.1016/j.ijsu.2021.105906 33789826

[37] Shea BJ Grimshaw JM Wells GA Boers M Andersson N Hamel C Development of AMSTAR: a measurement tool to assess the methodological quality of systematic reviews. BMC Med Res Methodol. 2007;7:10. 10.1186/1471-2288-7-10 17302989 PMC1810543

[38] Chen J Chen S Luo H Wu W Wang S The application of arsenic trioxide in cancer: An umbrella review of meta-analyses based on randomized controlled trials. J Ethnopharmacol. 2023;316:116734. 10.1016/j.jep.2023.116734 37290735

[39] Pérez-Bracchiglione J Meza N Bangdiwala SI Nino de Guzman E Urrutia G Bonfill X Graphical Representation of Overlap for OVErviews: GROOVE tool. Res Synth Methods. 2022;13:381–8. 10.1002/jrsm.1557 35278030

[40] Shea BJ Reeves BC Wells G Thuku M Hamel C Moran J AMSTAR 2: a critical appraisal tool for systematic reviews that include randomised or non-randomised studies of healthcare interventions, or both. BMJ. 2017;358:j4008. 10.1136/bmj.j4008 28935701 PMC5833365

[41] Guyatt GH Oxman AD Vist GE Kunz R Falck-Ytter Y Alonso-Coello P GRADE: an emerging consensus on rating quality of evidence and strength of recommendations. BMJ. 2008;336:924–6. 10.1136/bmj.39489.470347.AD 18436948 PMC2335261

[42] Kim S Park HJ Yang DH An intradialytic aerobic exercise program ameliorates frailty and improves dialysis adequacy and quality of life among hemodialysis patients: a randomized controlled trial. Kidney Res Clin Pract. 2022;41:462–72. 10.23876/j.krcp.21.284 35354243 PMC9346393

[43] Calvo-Lobo C Neyra-Bohorquez PP Seco-Calvo J Aerobic exercise effects in renal function and quality of life of patients with advanced chronic kidney disease. Rev Assoc Med Bras. 2019;65:657–62. 10.1590/1806-9282.65.5.657 31166442

[44] Andrade FP Rezende PdS Ferreira TdS Borba GC Müller AM Rovedder PME Effects of intradialytic exercise on cardiopulmonary capacity in chronic kidney disease: systematic review and meta-analysis of randomized clinical trials. Sci Rep. 2019;9:18470. 10.1038/s41598-019-54953-x 31804617 PMC6895108

[45] Liao MT Liu WC Lin FH Huang CF Chen SY Liu CC Intradialytic aerobic cycling exercise alleviates inflammation and improves endothelial progenitor cell count and bone density in hemodialysis patients. Medicine (Baltimore). 2016;95:e4134. 10.1097/MD.0000000000004134 27399127 PMC5058856

[46] Sovatzidis A Chatzinikolaou A Fatouros IG Panagoutsos S Draganidis D Nikolaidou E Intradialytic cardiovascular exercise training alters redox status, reduces inflammation and improves physical performance in patients with chronic kidney disease. Antioxidants. 2020;9:868. 10.3390/antiox9090868 32942555 PMC7554691

[47] Fuhro MI Dorneles GP Andrade FP Romão PR Peres A Monteiro MB Acute exercise during hemodialysis prevents the decrease in natural killer cells in patients with chronic kidney disease: a pilot study. Int Urol Nephrol. 2018;50:527–34. 10.1007/s11255-017-1747-z 29134614

[48] Oliveira E Silva VR Stringuetta Belik F Hueb JC de Souza Gonçalves R Costa Teixeira Caramori J Perez Vogt B Aerobic exercise training and nontraditional cardiovascular risk factors in hemodialysis patients: results from a prospective randomized trial. Cardiorenal Med. 2019;9:391–9. 10.1159/000501589 31597151

[49] March DS Lai KB Neal T Graham-Brown MP Highton PJ Churchward DR Circulating endotoxin and inflammation: associations with fitness, physical activity and the effect of a 6-month programme of cycling exercise during haemodialysis. Nephrol Dial Transplant. 2022;37:366–74. 10.1093/ndt/gfab178 33983449

[50] Liu YM Chung YC Chang JS Yeh ML Effects of aerobic exercise during hemodialysis on physical functional performance and depression. Biol Res Nurs. 2015;17:214–21. 10.1177/1099800414539548 25027035

[51] Perez-Dominguez B Suso-Marti L Dominguez-Navarro F Perpiña-Martinez S Calatayud J Casaña J Effects of resistance training on patients with End-Stage Renal Disease: an umbrella review with meta-analysis of the pooled findings. J Nephrol. 2023;36:1805–39. 10.1007/s40620-023-01635-7 37318646 PMC10543800

[52] Deus LA Corrêa HdL Neves RVP Reis AL Honorato FS Silva VL Are resistance training-induced BDNF in hemodialysis patients associated with depressive symptoms, quality of life, antioxidant capacity, and muscle strength? An insight for the muscle–brain–renal axis. Int J Environ Res Public Health. 2021;18:11299. 10.3390/ijerph182111299 34769814 PMC8583357

[53] Basir SS Mirzaei B Effects of moderate-intensity concurrent exercise training on cardiovascular risk factors in patients with chronic kidney disease undergoing hemodialysis: a randomized control trial. Sport Sci Health. 2022;18:1397–404. 10.1007/s11332-022-00911-6

[54] Frih B Jaafar H Mkacher W Ben Salah Z Hammami M Frih A The effect of interdialytic combined resistance and aerobic exercise training on health related outcomes in chronic hemodialysis patients: the Tunisian randomized controlled study. Front Physiol. 2017;8:288. 10.3389/fphys.2017.00288 28620308 PMC5449721

[55] Jia W He W Chen Z Wang H Lu H Determinants of dialysis adequacy in maintenance hemodialysis patients: a cross-sectional study on modifiable risk factors and clinical interventions. BMC Nephrol. 2025;26:369. 10.1186/s12882-025-04278-x 40634850 PMC12305990

[56] Khan MS Anker SD Friede T Jankowska EA Metra M Pina IL Minimal Clinically Important Differences in 6-Minute Walk Test in Patients With HFrEF and Iron Deficiency. J Card Fail. 2023;29:760–70. 10.1016/j.cardfail.2022.10.423 36332897

