# Supplementary material for: Effects of exercise on clinical outcomes in patients undergoing haemodialysis for chronic kidney disease: an umbrella review
Source: J Glob Health. 2026 Jul 24;16:04227. doi: 10.7189/jogh.16.04227 (PMC13397097; doi:10.7189/jogh.16.04227)
Supplement: Online Supplementary Document [file jogh-16-04227-s001.pdf]

Supplement to: Huang M, Xie Z, Lin B, Xiao W, Wen T, Li Y, Long H, Yang X, Zhang K. Effects of exercise on clinical outcomes in patients undergoing haemodialysis for chronic kidney disease: an umbrella review. J Glob Health. 2026;16:04227.

Text S1. Search strategy.

PubMed(42)

Search: (("Exercise Therapy"[MeSH Terms] OR "Rehabilitation Exercise" [Title/Abstract] OR "Exercise, Rehabilitation" [Title/Abstract] OR "Exercises, Rehabilitation" [Title/Abstract] OR "Rehabilitation Exercises" [Title/Abstract] OR "Therapy, Exercise" [Title/Abstract] OR "Exercise Therapies" [Title/Abstract] OR "Therapies, Exercise" [Title/Abstract] OR "Remedial Exercise" [Title/Abstract] OR "Exercise, Remedial" [Title/Abstract] OR "Remedial Exercises" [Title/Abstract] OR "Physical Activity" [Title/Abstract] OR "Exercise Therapy" [Title/Abstract] OR "Physical Rehabilitation" [Title/Abstract] OR "Physical Exercise" [Title/Abstract] OR "Fitness Training" [Title/Abstract] OR "Resistance Training" [Title/Abstract] OR "Aerobic Exercise" [Title/Abstract] OR "Strength Training" [Title/Abstract] OR "Endurance Exercise" [Title/Abstract] OR "Cardio Exercise" [Title/Abstract] OR "Movement Therapy" [Title/Abstract] OR "Therapeutic Exercise" [Title/Abstract] OR "Physiotherapy" [Title/Abstract] OR "Functional Training" [Title/Abstract] OR "Rehabilitation Program" [Title/Abstract] OR "Active Therapy" [Title/Abstract]) AND (("Hemodialysis, Home"[Mesh]) OR "Hemodialysis Units, Hospital"[Mesh] OR "Hemodialysis" [Title/Abstract] OR "Hemodialyses" [Title/Abstract] OR "Blood Dialysis" [Title/Abstract] OR "Hemofiltration" [Title/Abstract] OR "Intermittent Hemodialysis" [Title/Abstract] OR "Chronic Hemodialysis" [Title/Abstract] OR "Home Hemodialysis" [Title/Abstract] OR "Home Renal Dialysis" [Title/Abstract] OR "Hospital Hemodialysis Units" [Title/Abstract] OR "Hemodialysis Unit, Hospital" [Title/Abstract] OR "Hospital Hemodialysis Unit" [Title/Abstract])) AND ("Renal Insufficiency" [MeSH Terms] OR "Renal Insufficiencies" [Title/Abstract] OR "Kidney Insufficiency" [Title/Abstract] OR "Insufficiency, Kidney" [Title/Abstract] OR "Kidney Insufficiencies" [Title/Abstract] OR "Kidney Failure" [Title/Abstract] OR "Failure, Kidney" [Title/Abstract] OR "Failures, Kidney" [Title/Abstract] OR "Kidney Failures" [Title/Abstract] OR "Renal Failure" [Title/Abstract] OR "Failure, Renal" [Title/Abstract] OR "Failures,

Renal" [Title/Abstract] OR "Chronic Kidney Disease" [Title/Abstract] OR "CKD" [Title/Abstract] OR "End-Stage Renal Disease" [Title/Abstract] OR "ESRD" [Title/Abstract] OR "Chronic Renal Insufficiency" [Title/Abstract] OR "Kidney Dysfunction" [Title/Abstract] OR "End-Stage Kidney Disease" [Title/Abstract] OR "ESKD" [Title/Abstract] OR "Acute Kidney Failure" [Title/Abstract] OR "Uremia" [Title/Abstract] OR "End-Stage Renal Insufficiency" [Title/Abstract] OR "Nephropathy" [Title/Abstract]) Filters: Meta-Analysis, Systematic Review

Embase(95)

Embase
Session Results
....

No.	Query	Results
#11.	#10 AND ('meta analysis'/de OR 'systematic review'/de)	95
#10.	#3 AND #6 AND #9	1,287
#9.	#7 OR #8	734,918
#8.	'renal insufficiencies':ab,kw,ti OR 'kidney insufficiency':ab,kw,ti OR 'insufficiency, kidney':ab,kw,ti OR 'kidney insufficiencies':ab,kw,ti OR 'kidney failure':ab,kw,ti OR 'failure, kidney':ab,kw,ti OR 'failures, kidney':ab,kw,ti OR 'kidney failures':ab,kw,ti OR 'renal failure':ab,kw,ti OR 'failure, renal':ab,kw,ti OR 'failures, renal':ab,kw,ti OR 'chronic kidney disease':ab,kw,ti OR 'ckd':ab,kw,ti OR 'end-stage renal disease':ab,kw,ti OR 'esrd':ab,kw,ti OR 'chronic renal insufficiency':ab,kw,ti OR 'kidney dysfunction':ab,kw,ti OR 'end-stage kidney disease':ab,kw,ti OR 'eskd':ab,kw,ti OR 'acute kidney failure':ab,kw,ti OR 'uremia':ab,kw,ti OR 'end-stage renal insufficiency':ab,kw,ti OR 'nephropathy':ab,kw,ti	467,531
#7.	'kidney failure'/exp	585,844
#6.	#4 OR #5	204,734
#5.	'hemodialysis':ab,kw,ti OR 'hemodialyses':ab,kw,ti OR 'blood dialysis':ab,kw,ti OR 'hemofiltration':ab,kw,ti OR 'intermittent hemodialysis':ab,kw,ti OR 'chronic hemodialysis':ab,kw,ti OR 'home hemodialysis':ab,kw,ti OR 'home renal dialysis':ab,kw,ti OR 'hospital hemodialysis units':ab,kw,ti OR 'hemodialysis unit, hospital':ab,kw,ti OR 'hospital hemodialysis unit':ab,kw,ti	124,064
#4.	'hemodialysis'/exp OR 'hemodialysis'	200,848
#3.	#1 OR #2	441,352
#2.	'rehabilitation exercise':ab,kw,ti OR 'exercise, rehabilitation':ab,kw,ti OR 'exercises, rehabilitation':ab,kw,ti OR 'rehabilitation exercises':ab,kw,ti OR 'therapy, exercise':ab,kw,ti OR 'exercise	363,085

therapies':ab,kw,ti OR 'therapies, exercise':ab,kw,ti OR 'remedial exercise':ab,kw,ti OR 'exercise, remedial':ab,kw,ti OR 'remedial exercises':ab,kw,ti OR 'physical activity':ab,kw,ti OR 'exercise therapy':ab,kw,ti OR 'physical rehabilitation':ab,kw,ti OR 'physical exercise':ab,kw,ti OR 'fitness training':ab,kw,ti OR 'resistance training':ab,kw,ti OR 'aerobic exercise':ab,kw,ti OR 'strength training':ab,kw,ti OR 'endurance exercise':ab,kw,ti OR 'cardio exercise':ab,kw,ti OR 'movement therapy':ab,kw,ti OR 'therapeutic exercise':ab,kw,ti OR 'physiotherapy':ab,kw,ti OR 'functional training':ab,kw,ti OR 'rehabilitation program':ab,kw,ti OR 'active therapy':ab,kw,ti

#1. 'kinesiotherapy'/exp OR 'kinesiotherapy' 111,194

....

Cochrane(245)

ID Search Hits

#1 MeSH descriptor: [undefined] explode all trees 0

#2 'Rehabilitation Exercise' OR 'Exercise, Rehabilitation' OR 'Exercises, Rehabilitation' OR 'Rehabilitation Exercises' OR 'Therapy, Exercise' OR 'Exercise Therapies' OR 'Therapies, Exercise' OR 'Remedial Exercise' OR 'Exercise, Remedial' OR 'Remedial Exercises' OR 'Physical Activity' OR 'Exercise Therapy' OR 'Physical Rehabilitation' OR 'Physical Exercise' OR 'Fitness Training' OR 'Resistance Training' OR 'Aerobic Exercise' OR 'Strength Training' OR 'Endurance Exercise' OR 'Cardio Exercise' OR 'Movement Therapy' OR 'Therapeutic Exercise' OR 'Physiotherapy' OR 'Functional Training' OR 'Rehabilitation Program' OR 'Active Therapy' 270415

#3 #1 OR #2 270415

#4 MeSH descriptor: [Hemodialysis Units, Hospital] explode all trees 15

#5 MeSH descriptor: [Hemodialysis, Home] explode all trees 65

#6 'Hemodialysis' OR 'Hemodialyses' OR 'Blood Dialysis' OR 'Hemofiltration' OR 'Intermittent Hemodialysis' OR 'Chronic Hemodialysis' OR 'Home Hemodialysis' OR 'Home Renal Dialysis' OR 'Hospital Hemodialysis Units' OR 'Hemodialysis Unit, Hospital' OR 'Hospital Hemodialysis Unit' 20733

#7 #4 OR #5 OR #620733

#8 MeSH descriptor: [Renal Insufficiency] explode all trees 13366

#9 'Renal Insufficiencies' OR 'Kidney Insufficiency' OR 'Insufficiency, Kidney' OR 'Kidney Insufficiencies' OR 'Kidney Failure' OR 'Failure, Kidney' OR 'Failures, Kidney' OR 'Kidney Failures' OR 'Renal Failure' OR 'Failure, Renal' OR 'Failures, Renal' OR 'Chronic Kidney Disease' OR 'CKD' OR 'End-Stage Renal Disease' OR 'ESRD' OR 'Chronic Renal Insufficiency' OR 'Kidney Dysfunction' OR 'End-Stage Kidney Disease' OR 'ESKD' OR 'Acute Kidney Failure' OR 'Uremia' OR 'End-Stage Renal Insufficiency' OR 'Nephropathy' 57722

#10 #8 OR #9 58716

#11 #3 AND #7 AND #10 1543

Web of Science(307)

Searches:

1: TS=(Exercise intervention) OR TS=(Exercise Therapy) OR TS=(Rehabilitation Exercise) OR TS=(Exercise, Rehabilitation) OR TS=(Exercises, Rehabilitation) OR TS=(Rehabilitation Exercises) OR TS=(Therapy,

Exercise) OR TS=(Exercise Therapies) OR TS=(Therapies, Exercise) OR TS=(Remedial Exercise) OR TS=(Exercise, Remedial) OR TS=(Remedial Exercises) OR TS=(Physical Activity) OR TS=(Exercise Therapy) OR TS=(Physical Rehabilitation) OR TS=(Physical Exercise) OR TS=(Fitness Training) OR TS=(Resistance Training) OR TS=(Aerobic Exercise) OR TS=(Strength Training) OR TS=(Endurance Exercise) OR TS=(Cardio Exercise) OR TS=(Movement Therapy) OR TS=(Therapeutic Exercise) OR TS=(Physiotherapy) OR TS=(Functional Training) OR TS=(Rehabilitation Program) OR TS=(Active Therapy) and Preprint Citation Index (Exclude – Database) Results: 2916165

2: TS=(Hemodialysis, Home) OR TS=(Hemodialysis Units, Hospital) OR TS=(Hemodialysis) OR TS=(Hemodialyses) OR TS=(Blood Dialysis) OR TS=(Hemofiltration) OR TS=(Intermittent Hemodialysis) OR TS=(Chronic Hemodialysis) OR TS=(Home Hemodialysis) OR TS=(Home Renal Dialysis) OR TS=(Hospital Hemodialysis Units) OR TS=(Hemodialysis Unit, Hospital) OR TS=(Hospital Hemodialysis Unit) and Preprint Citation Index (Exclude – Database) Results: 213517

3: TS=(Renal Insufficiency) OR TS=(Renal Insufficiencies) OR TS=(Kidney Insufficiency) OR TS=(Insufficiency, Kidney) OR TS=(Kidney Insufficiencies) OR TS=(Kidney Failure) OR TS=(Failure, Kidney) OR TS=(Failures, Kidney) OR TS=(Kidney Failures) OR TS=(Renal Failure) OR TS=(Failure, Renal) OR TS=(Failures, Renal) OR TS=(Chronic Kidney Disease) OR TS=(CKD) OR TS=(End-Stage Renal Disease) OR TS=(ESRD) OR TS=(Chronic Renal Insufficiency) OR TS=(Kidney Dysfunction) OR TS=(End-Stage Kidney Disease) OR TS=(ESKD) OR TS=(Acute Kidney Failure) OR TS=(Uremia) OR TS=(End-Stage Renal Insufficiency) OR TS=(Nephropathy) and Preprint Citation Index (Exclude – Database)
Results: 737324

4: #1 AND #2 AND #3 and Preprint Citation Index (Exclude – Database)
Results: 11488

5: TS=(systematic review) OR TS=(meta-analysis) and Preprint Citation Index (Exclude – Database)
Results: 825067

6: #4 AND #5 and Preprint Citation Index (Exclude – Database) Results: 307

Figure S1. Overlap.

1. KT/v

r=19, N=40, c=5

CCA= (40-19)/(19×5-19) = 21/76 = **27.6%→Very high**

Reference	Sheng 2014	Salhab 2019	Ferreira 201	Huang 2019	Ferrari 2020
Afshar, 2010	1	1	1	1	1
Cheema, 2007				1	
Dobsak, 2012		1	1	1	1
Parsons, 2004		1		1	1
Pellizzaro, 2013				1	
Reboredo, 2010	1		1	1	1
van Vilsteren, 2005	1			1	1
Giannaki, 2013	1		1		1
Parsons, 2006	1				
Mohseni, 2013	1		1		1
Adam 2017			1		
Groussard 2015		1	1		1
Kopple 2007			1		
Liao 2016			1		1
van Vilsteren 2004			1		
Sakkas 2008		1			
Fernandes 2019					1
Hristea 2016					1
Paluchamy 2018					1

	Sheng 2014	Salhab 2019	Ferreira 2019	Huang 2019
Salhab 2019	10.0%			
Ferreira 2019	33.3%	25.0%		
Huang 2019	30.0%	33.3%	21.4%	
Ferrari 2020	38.5%	30.8%	46.7%	35.7%

2. VO2 peak

r=15, N=40, c=6

CCA= (40-15)/(15×6-15) = 25/75 = **33.3%→Very high**

Reference	Sheng 2014	Chung 2017	Young 2018	Huang 2019	Ferrari 202	Bogataj 202
Van Vilsteren, 2005	1	1		1		
Koufaki, 2002	1					
Painter, 2002	1	1	1			1
Petraki, 2008	1				1	
Ouzouni, 2009	1	1		1	1	1
Konstantinidou, 200	1			1	1	1
Kouidi, 2009	1	1		1	1	1
Bohm 2014		1				1
Reboredo 2011		1	1	1		1
Carmack 1995			1			1
Groussard 2015			1	1		1
Deligiannis, 1999				1		1
Goldberg, 1986				1		
Tsuyuki						1
Danillidis						1

	Sheng 2014	Chung 2017	Young 2018	Huang 2019	Ferrari 2020
Chung 2017	44.4%				
Young 2018	10.0%	25.0%			
Huang 2019	36.4%	40.0%	20.0%		
Ferrari 2020	57.1%	25.0%	0.0%	33.3%	
Bogataj 2020	28.6%	41.7%	36.4%	46.2%	25.0%

3. 6MWT

r=15, N=36, c=6

CCA= (36-15)/(15×6-15) = 21/75 = **28.0%→Very high**

Reference	Sheng 2014	Chung 2017	Young 2018	Huang 2019	Ferrari 202	Bogataj 202
Koh 2010	1	1	1	1		1
Pellizzaro 2013	1			1		1
Cheema 2007	1	1		1		1
DePaul 2002	1			1	1	1
Bohm 2014		1				1
Dobsak 2012		1		1	1	1
Groussard 2015			1	1	1	1
Fernandes 2019					1	
Hristea 2016					1	1
Wu 2014					1	1
Liao						1
Kirkman						1
Orcy						1
Segura-Orti						1
Frih						1

	Sheng 2014	Chung 2017	Young 2018	Huang 2019	Ferrari 2020
Chung 2017	33.3%				
Young 2018	20.0%	20.0%			
Huang 2019	66.7%	42.9%	33.3%		
Ferrari 2020	11.1%	11.1%	14.3%	33.3%	
Bogataj 2020	28.6%	28.6%	14.3%	42.9%	33.3%

4. SBP

r=14, N=23, c=4

CCA= (23-14)/(14×4-14) = 9/42 = **21.4%→Very high**

Reference	Sheng 2014	Young 2018	Huang 2019	Ferrari 202
Koh 2010	1	1	1	
Petraki 2008	1			
Ouzouni 2009	1		1	
Toussaint 2008	1	1	1	1
Wilund 2010	1			1
DePaul 2002	1			
Van Vilsteren 2005	1		1	1
Deligiannis, 1999			1	
Cooke 2018				1
Fernandes 2019				1
Liao 2016				1
Painter 2002				1
Pulachamy 2018				1
Soliman 2015				1

	Sheng 2014	Young 2018	Huang 2019
Young 2018	28.6%		
Huang 2019	50.0%	40.0%	
Ferrari 2020	23.1%	10.0%	16.7%

5. DBP

r=15, N=25, c=4

CCA= (25-15)/(15×4-15) = 10/45 = **22.2%→Very high**

Reference	Sheng 2014	Young 2018	Huang 2019	Ferrari 2020
Koh 2010	1	1	1	
Petraki 2008	1			
Ouzouni 2009	1		1	
Toussaint 2008	1	1	1	1
Wilund 2010	1	1		1
DePaul 2002	1			
Van Vilsteren 2005	1		1	1
Dobsak 2012			1	
Deligiannis, 1999			1	
Fernandes 2019				1
Liao 2016				1
Painter 2002				1
Pulachamy 2018				1
Soliman 2015				1
Cooke 2018				1

	Sheng 2014	Young 2018	Huang 2019
Young 2018	42.9%		
Huang 2019	44.4%	28.6%	
Ferrari 2020	23.1%	20.0%	15.4%

6. PCS

r=12, N=28, c=6

CCA= (28-12)/(12×6-12) = 16/60 = **26.7%→Very high**

Reference	Sheng 2014	Chung 2017	Young 2018	Huang 2019	Salhab 2019	Molsted 201
Koh, 2010	1	1	1	1	1	
Giannaki, 2013	1		1		1	
Song, 2012	1			1		1
Johansen, 2006	1	1				
Chen, 2010	1					1
Ouzouni, 2009	1	1		1		
DePaul, 2002	1					
Dobsak 2012		1		1	1	
Parsons 2004		1		1		
van Vilsteren 2004		1		1		
Sakkas 2008					1	
Painter 2000					1	

	Sheng 2014	Chung 2017	Young 2018	Huang 2019	Salhab 2019
Chung 2017	30.0%				
Young 2018	28.6%	14.3%			
Huang 2019	30.0%	71.4%	14.3%		
Salhab 2019	20.0%	22.2%	40.0%	22.2%	
Molsted 2019	28.6%	0.0%	0.0%	14.3%	0.0%

7. MCS

r=12, N=18, c=4

CCA= (18-12)/(12×4-12) = 6/36 = **16.7%→Very high**

Reference	Chung 2017	Young 2018	Huang 2019	Salhab 201
Koh, 2010	1	1	1	1
Giannaki, 2013		1		1
Song, 2012			1	
Johansen, 2006				
Chen, 2010				
Ouzouni, 2009	1		1	
DePaul, 2002				
Dobsak 2012	1		1	1
Parsons 2004	1		1	
van Vilsteren 2004	1		1	
Sakkas 2008				1
Painter 2000				1

	Chung 2017	Young 2018	Huang 2019
Young 2018	16.7%		
Huang 2019	83.3%	14.3%	
Salhab 2019	25.0%	40.0%	22.2%

8. CRP

r=9, N=12, c=2

CCA= (12-9)/(9×2-9) = 3/9 = **33.3%→Very high**

Reference	Bogataj 2020	Ferrari 202
Afshar 2010	1	1
Hristea 2016	1	1
Liao 2016	1	1
Cheema	1	
Kopple	1	
Abreu	1	
Fuhro 2017		1
Suhardjono 2019		1
Toussaint 2008		1

	Bogataj 2020
Ferrari 2020	33.3%

9. serum phosphorus

r=4, N=5, c=2

CCA= (5-4)/(4×2-4) = 1/4 = **25.0%→Very high**

Reference	Ferreira 201	Salhab 2019
Makhlough 2012	1	1
Reboredo 2010	1	
Wilund 2010		1
De Lima 2013		1

	Ferreira 2019
Salhab 2019	25.0%

10. Depression

r=13, N=15, c=2

CCA= (15-13)/(13×2-13) = 2/13 = **15.4%→Very high**

Reference	Chung 201	Yu 2024
Giannaki 2013	1	
Kouidi 2010	1	1
Ouzouni 2009	1	1
van Vilsteren 2004	1	
Lin 2021		1
Zhou 2020		1
Yabe 2022		1
Sakkas 2008		1
Zhou 2023		1
Rhee 2019		1
Carney 1987		1
Cheema 2006		1
Liu 2015		1

	Chung 2017
Yu 2024	15.4%

Table S1. Excluded studies

Excluded studies	Reason for exclusion
[1]	Inappropriate comparison
[2]	Inappropriate interventions
[3]	Inappropriate interventions
[4]	Inappropriate interventions
[5]	Inappropriate interventions
[6]	Inappropriate interventions
[7]	Inappropriate interventions
[8]	Inappropriate outcomes
[9]	Inappropriate outcomes
[10]	Inappropriate outcomes
[11]	Inappropriate outcomes
[12]	Inappropriate outcomes
[13]	Inappropriate outcomes
[14]	Inappropriate outcomes
[15]	Inappropriate outcomes
[16]	Incomplete data
[17]	Incomplete data
[18]	Incomplete data
[19]	Incomplete data

Table S2. Outcome level GRADE assessment for each exercise modality

Outcome	Exercise modality	Contributing MAs	Downgrade					Upgrade	Final certainty
			**Risk of bias**	**Inconsistency**	**Indirectness**	**Imprecision**	**Publication bias**		
Kt/V	Aerobic	Sheng[20], Salhab[21], Ferreira[22], Ferrari[23]	−1	−1	0	0	−1	0	**Low**
Kt/V	Resistance	Ferrari[23]	−1	0	0	−1	0	0	**Low**
Kt/V	Combined	Sheng[20], Huang[24]	−1	0	0	0	0	0	**Low**
VO_2 peak	Aerobic	Young[25], Huang[24], Ferrari[23]	−1	0	0	0	−1	0	**Low**
VO_2 peak	Combined	Sheng[20], Chung[26], Huang[24], Ferrari[23], Bogataj[27]	−1	0	0	0	0	+1	**Moderate**
6MWT	Aerobic	Young[25], Huang[24], Ferrari[23]	−1	0	0	0	−1	+1	**Low**
6MWT	Resistance	Huang[24], Ferrari[23]	−1	−1	0	0	0	0	**Low**
6MWT	Combined	Sheng[20], Chung[26], Huang[24],	−1	0	0	−1	0	0	**Very low**

Outcome	Exercise modality	Contributing MAs	Downgrade					Upgrade	Final certainty
			Risk of bias	Inconsistency	Indirectness	Imprecision	Publication bias		
		Ferrari[23], Bogataj[27]							
SBP	Aerobic	Young[25], Huang[24], Ferrari[23]	−1	−1	0	0	−1	0	**Very low**
SBP	Combined	Sheng[20], Huang[24], Ferrari[23]	−1	0	0	−1	0	0	**Low**
DBP	Aerobic	Young[25], Huang[24], Ferrari[23]	−1	−1	0	−1	0	0	**Very low**
DBP	Combined	Sheng[20], Huang[24], Ferrari[23]	−1	0	0	0	0	0	**Low**
PCS	Aerobic	Young[25], Salhab[21], Huang[24]	−1	0	0	−1	0	0	**Low**
PCS	Resistance	Molsted[28]	−1	0	0	0	−1	0	**Low**
PCS	Combined	Sheng[20], Chung[26], Huang[24]	−1	0	0	0	0	0	**Low**
MCS	Aerobic	Young[25], Salhab[21]	−1	0	0	−1	0	0	**Low**

Outcome	Exercise modality	Contributing MAs	Downgrade					Upgrade	Final certainty
			Risk of bias	Inconsistency	Indirectness	Imprecision	Publication bias		
MCS	Combined	Chung[26], Huang[24]	−1	0	0	0	0	0	**Low**
CRP	Aerobic	Ferrari[23]	−1	0	0	0	−1	+1	**Low**
CRP	Resistance	Ferrari[23]	−1	0	0	−1	0	0	**Very low**
CRP	Combined	Bogataj[27], Ferrari[23]	−1	0	0	0	0	+1	**Moderate**
Depression	Aerobic	Yu[29]	−1	0	0	0	−1	+1	**Low**
Depression	Resistance	Yu[29]	−1	0	0	−1	0	0	**Very low**
Depression	Combined	Chung[26], Yu[29]	−1	−1	0	0	0	0	**Low**
Serum phosphorus	Aerobic	Salhab[21], Ferreira[22]	−1	−1	0	−1	0	0	**Very low**

Abbreviations: Not available (NA); Non-significant (NS); Aerobic training (AT); Resistance training (RT); Combined training (CT); Meta-analysis (MA); Weighted mean difference (WMD); Standardized mean difference (SMD); Mean difference (MD); Confidence interval (CI); Urea clearance index (Kt/V); Peak oxygen consumption (VO_2 peak); Short-form 36 health questionnaire (SF-36); 6-minute walk test (6MWT); Physical component score (PCS); Mental component score (MCS); Systolic blood pressure (SBP); Diastolic blood pressure (DBP); C-reactive protein (CRP); A Measurement Tool to Assess Systematic Reviews 2 (AMSTAR 2).

Table S3: Summary of effect estimates and certainty of evidence for each exercise modality

Outcome	Exercise modality	95% CI	Effect measure	P-value	I^2	GRADE	Evidence class	Reason for selection
Kt/V	Aerobic	0.08 (0.00–0.15)	WMD	0.04	56	Low	IV	Highest AMSTAR 2; largest sample size (50 studies)
Kt/V	Resistance	0.10 (–0.01–0.20)	WMD	0.06	0	Low	NS	only MA reporting resistance Kt/V with low heterogeneity
Kt/V	Combined	0.19 (–0.06–0.43)	SMD	0.14	0	Low	NS	Only MA analyzing combined training for Kt/V; low heterogeneity
VO_2 peak	Aerobic	2.07 (0.42–3.72)	WMD	0.01	0	Low	IV	Highest AMSTAR 2; most recent; no heterogeneity
VO_2 peak	Combined	5.41 (4.03–6.79)	WMD	<0.00001	0	Moderate	III	largest effect size with zero heterogeneity
6MWT	Aerobic	64.98 (43.86–86.11)	WMD	<0.00001	0	Low	III	Highest AMSTAR 2; largest number of RCTs; moderate certainty
6MWT	Resistance	68.50 (29.05–107.96)	WMD	0.0007	36	Low	III	Only MA with resistance-specific 6MWT data; moderate heterogeneity
6MWT	Combined	36.37 (–13.73–86.46)	WMD	0.15	0	Very low	NS	Consistent with priority rule for all 6MWT outcomes

SBP	Aerobic	–10.07 (–16.35 to –3.78)	WMD	0.002	44	Very low	IV	Highest AMSTAR 2; clear effect estimate
SBP	Combined	–4.33 (–9.75–1.08)	WMD	0.12	0	Low	NS	Only combined SBP data available
DBP	Aerobic	–2.96 (–7.71–1.78)	WMD	0.22	65	Very low	NS	Highest AMSTAR 2; acknowledges high heterogeneity
DBP	Combined	–5.76 (–8.83 to –2.70)	WMD	0.0002	0	Low	III	Only combined DBP estimate with zero heterogeneity
PCS	Aerobic	1.97 (–8.27–12.22)	MD	0.71	18	Low	NS	Only aerobic PCS MA with low heterogeneity
PCS	Resistance	10.05 (2.95–17.14)	MD	0.0006	0	Low	IV	Only MA reporting resistance-only PCS; zero heterogeneity
PCS	Combined	0.34 (0.09–0.59)	SMD	0.007	27	Low	IV	Most comprehensive combined-training MA; low heterogeneity
MCS	Aerobic	3.37 (–7.94–14.68)	MD	0.56	0	Low	NS	Only aerobic MCS MA with precise estimate; zero heterogeneity
MCS	Combined	0.27 (0.02–0.51)	SMD	0.03	0	Low	IV	Only combined MCS data; statistically significant
CRP	Aerobic	–3.28 (–4.68 to –1.88)	WMD	<0.00001	0	Low	III	Highest AMSTAR 2; largest

								sample; zero heterogeneity
CRP	Resistance	–0.50 (–1.52–0.52)	WMD	0.34	10	Very low	NS	Only resistance CRP estimate; low heterogeneity
CRP	Combined	–0.82 (–1.04 to –0.60)	SMD	<0.001	NA	Moderate	III	Only combined-training CRP MA; significant effect
Depression	Aerobic	–0.93 (–1.32 to –0.55)	SMD	<0.001	0	Low	III	Most recent; largest number of RCTs; zero heterogeneity
Depression	Resistance	–0.40 (–0.96–0.17)	SMD	1.00	0	Very low	NS	Only MA with resistance-only depression data; precise null effect
Depression	Combined	–0.85 (–1.29 to –0.41)	SMD	<0.001	76.07	Low	III	Most recent and comprehensive; heterogeneity accepted due to clinical diversity
Serum phosphorus	Aerobic	Not significant		NS	–	Very low	NS	Only MA providing full MD with CI; very high heterogeneity noted

Abbreviations: Not available (NA); Non-Significant (NS); Aerobic training (AT); Resistance training (RT), Combined training (CT); Urea Clearance Index (Kt/V); Peak oxygen consumption (VO_2 peak); Short-form 36 health questionnaire (SF-36); 6-minute walk test (6MWT); Physical component scores (PCS); Mental component scores (MCS); Systolic blood pressure (SBP); Diastolic blood pressure (DBP); C-reactive protein (CRP); Standardized Mean Difference (SMD); Mean Deviation (MD).

Reference:

1. Kowal, G. and A. Rydzewski, *The effects of respiratory muscle training in chronic kidney disease patients on haemodialysis and peritoneal dialysis: A review.* Medical Studies/Studia Medyczne, 2018. **34**(1): p. 78-85.
2. Yang, B., et al., *Non-pharmacological interventions for improving sleep quality in patients on dialysis: Systematic review and meta-analysis.* Sleep Medicine Reviews, 2015. **23**: p. 68-82.
3. Li, H., et al., *Effects of nonpharmacological intervention on sleep quality in hemodialysis patients A protocol for systematic review and meta-analysis.* Medicine, 2021. **100**(27).
4. Almanza, A., et al., *Effects of physical training with vascular flow restriction during hemodialysis.* Revista de Nefrologia, Dialisis y Trasplante, 2022. **42**(1): p. 11-21.
5. Kesik, G. and N. Altinok Ersoy, *The effect of nonpharmacologic interventions for muscle cramps and restless-leg syndrome in hemodialysis patients: A meta-analysis of randomized controlled trials.* Therapeutic Apheresis and Dialysis, 2023. **27**(4): p. 636-654.
6. Brüggemann, A.K.V., et al., *Inspiratory Muscle Training in Patients Living With Chronic Kidney Disease and Receiving Hemodialysis: Meta-Analysis of Randomized Controlled Trials.* Physical therapy, 2024. **104**(8).
7. Verrelli, D., et al., *Effect of Intradialytic Exercise on Cardiovascular Outcomes in Maintenance Hemodialysis: A Systematic Review and Meta-Analysis.* Kidney360, 2024. **5**(3): p. 390-413.
8. Clarkson, M.T., et al., *Exercise interventions for improving objective physical function in patients with end-stage kidney disease on dialysis: a systematic review and meta-analysis.* American Journal of Physiology-Renal Physiology, 2019. **316**(5): p. F856-F872.
9. Lu, Y., Y. Wang, and Q. Lu, *Effects of Exercise on Muscle Fitness in Dialysis Patients: A Sys tematic Review and Meta-Analysis.* American Journal of Nephrology, 2019. **50**(4): p. 291-302.
10. Sarmiento Becerra, O.M., A.M. Puentes Salazar, and A.E. Hernández, *¿Cuál es la seguridad de un programa de ejercicio, como intervención, durante la hemodiálisis para el paciente con enfermedad renal crónica?* Revista Colombiana de Nefrología, 2019. **6**(1): p. 35-47.
11. Scapini, K.B., et al., *Combined training is the most effective training modality to improve aerobic capacity and blood pressure control in people requiring haemodialysis for end-stage renal disease: systematic review and network meta-analysis.* Journal of physiotherapy, 2019. **65**(1): p. 4-15.
12. Hargrove, N., et al., *Effect of Aerobic Exercise on Dialysis-Related Symptoms in Individuals Undergoing Maintenance Hemodialysis: A Systematic Review and Meta-Analysis of Clinical Trials.* Clin J Am Soc Nephrol, 2021. **16**(4): p. 560-574.
13. Andrade, F.P., et al., *Effects of upper limb exercise programs on the arteriovenous fistula in patients on hemodialysis: A systematic review and meta-analysis.* J Vasc Access, 2022. **23**(5): p. 770-777.
14. Li, Y., X. Wang, and Z. Pei, *Advances in Exercise Therapy in Hemodialysis: A Systematic Review.* Mini Rev Med Chem, 2023. **23**(18): p. 1838-1844.
15. Wahida, A.Z., H. Rumahorbo, and Murtiningsih, *The effectiveness of intradialytic exercise in ameliorating fatigue symptoms in patients with chronic kidney failure undergoing hemodialysis: A systematic literature review and meta-analysis.* J Taibah Univ Med Sci, 2023. **18**(3): p. 512-525.
16. Bakaloudi, D.R., et al., *The Effect of Exercise on Nutritional Status and Body Composition in Hemodialysis: A Systematic Review.* Nutrients, 2020. **12**(10).
17. Bogataj, Š., et al., *Physical exercise and cognitive training interventions to improve cognition in hemodialysis patients: A systematic review.* Front Public Health, 2022. **10**: p. 1032076.

18. Meléndez Oliva, E., et al., *Effect of Exercise on Inflammation in Hemodialysis Patients: A Systematic Review.* J Pers Med, 2022. **12**(7).
19. Zang, W., et al., *Comparative efficacy of exercise modalities for cardiopulmonary function in hemodialysis patients: A systematic review and network meta-analysis.* Front Public Health, 2022. **10**: p. 1040704.
20. Sheng, K., et al., *Intradialytic Exercise in Hemodialysis Patients: A Systematic Review and Meta-Analysis.* American Journal of Nephrology, 2014. **40**(5): p. 478-490.
21. Salhab, N., et al., *Effects of intradialytic aerobic exercise on hemodialysis patients: a systematic review and meta-analysis.* J Nephrol, 2019. **32**(4): p. 549-566.
22. Ferreira, G.D., et al., *Does Intradialytic Exercise Improve Removal of Solutes by Hemodialysis? A Systematic Review and Meta-analysis.* Arch Phys Med Rehabil, 2019. **100**(12): p. 2371-2380.
23. Ferrari, F., et al., *Intradialytic training in patients with end-stage renal disease: a systematic review and meta-analysis of randomized clinical trials assessing the effects of five different training interventions.* Journal of Nephrology, 2020. **33**(2): p. 251-266.
24. Huang, M., et al., *Exercise Training and Outcomes in Hemodialysis Patients: Systematic Review and Meta-Analysis.* Am J Nephrol, 2019. **50**(4): p. 240-254.
25. Young, H.M.L., et al., *Effects of intradialytic cycling exercise on exercise capacity, quality of life, physical function and cardiovascular measures in adult haemodialysis patients: a systematic review and meta-analysis.* Nephrology Dialysis Transplantation, 2018. **33**(8): p. 1436-1445.
26. Chung, Y.C., M.L. Yeh, and Y.M. Liu, *Effects of intradialytic exercise on the physical function, depression and quality of life for haemodialysis patients: a systematic review and meta-analysis of randomised controlled trials.* Journal of clinical nursing, 2017. **26**(13-14): p. 1801-1813.
27. Bogataj, Š., et al., *Exercise-based interventions in hemodialysis patients: A systematic review with a meta-analysis of randomized controlled trials.* Journal of Clinical Medicine, 2020. **9**(1).
28. Molsted, S., A.S.D. Bjørkman, and L.H. Lundstrøm, *Effects of strength training to patients undergoing dialysis: A systematic review.* Danish Medical Journal, 2019. **66**(1).
29. Yu, H., et al., *The effects of exercise training interventions on depression in hemodialysis patients.* Frontiers in Psychiatry, 2024. **14**.
